# Cost‐effectiveness of intensive care for hospitalized COVID-19 patients: experience from South Africa

**DOI:** 10.1186/s12913-021-06081-4

**Published:** 2021-01-22

**Authors:** S. M. Cleary, T. Wilkinson, C. R. Tamandjou Tchuem, S. Docrat, G. C. Solanki

**Affiliations:** 1 Health Economics Unit, School of Public Health and Family Medicine, University of Cape Town, Cape Town, South Africa; 2 Health Systems Research Unit, South African Medical Research Council, Cape Town, South Africa; 3 NMG Consultants and Actuaries, Cape Town, South Africa

**Keywords:** COVID-19, SARS-CoV-2, Cost‐effectiveness, Intensive care unit, Priority setting, South Africa

## Abstract

**Background:**

Given projected shortages of critical care capacity in public hospitals during the COVID-19 pandemic, the South African government embarked on an initiative to purchase this capacity from private hospitals. In order to inform purchasing decisions, we assessed the cost-effectiveness of intensive care management for admitted COVID-19 patients across the public and private health systems in South Africa.

**Methods:**

Using a modelling framework and health system perspective, costs and health outcomes of inpatient management of severe and critical COVID-19 patients in (1) general ward and intensive care (GW + ICU) versus (2) general ward only (GW) were assessed. Disability adjusted life years (DALYs) were evaluated and the cost per admission in public and private sectors was determined. The model made use of four variables: mortality rates, utilisation of inpatient days for each management approach, disability weights associated with severity of disease, and the unit cost per general ward day and per ICU day in public and private hospitals. Unit costs were multiplied by utilisation estimates to determine the cost per admission. DALYs were calculated as the sum of years of life lost (YLL) and years lived with disability (YLD). An incremental cost-effectiveness ratio (ICER) - representing difference in costs and health outcomes of the two management strategies - was compared to a cost-effectiveness threshold to determine the value for money of expansion in ICU services during COVID-19 surges.

**Results:**

A cost per admission of ZAR 75,127 was estimated for inpatient management of severe and critical COVID-19 patients in GW as opposed to ZAR 103,030 in GW + ICU. DALYs were 1.48 and 1.10 in GW versus GW + ICU, respectively. The ratio of difference in costs and health outcomes between the two management strategies produced an ICER of ZAR 73,091 per DALY averted, a value above the cost-effectiveness threshold of ZAR 38,465.

**Conclusions:**

Results indicated that purchasing ICU capacity from the private sector during COVID-19 surges may not be a cost-effective investment. The ‘real time’, rapid, pragmatic, and transparent nature of this analysis demonstrates an approach for evidence generation for decision making relating to the COVID-19 pandemic response and South Africa’s wider priority setting agenda.

## Background

The COVID-19 pandemic has intensified demands on the health care system and resulted in critical shortages of resources (hospital beds, intensive care unit (ICU) beds, ventilators, medical workforce), particularly in the South African public sector. A major area of concern, globally and in South Africa, was the sufficiency of ICU capacity for the management of critically ill COVID-19 patients. Against an ICU bed availability of 3,318 (1,178 public and 2,140 private), the South African Portfolio Committee on Health highlighted a shortfall in ICU beds in the country; where the peak daily demand for ICU beds was projected to be between 4,100 beds (optimistic scenario) and 14,767 (pessimistic scenario) [1]. Based on the expected progression of COVID-19 in South Africa and expected utilisation rates of ICU in the management of COVID-19, projections suggested insufficient supply of ICU capacity in the public sector. Government adopted a two-pronged strategy including (1) the adoption of a lockdown strategy to flatten the curve in an effort to reduce the likelihood of demand exceeding the available health care supply and (2) initiatives to purchase critical bed capacity from the South African private sector for use by public sector patients.

Intensive care services are very expensive and are one of the largest drivers of hospital costs, even in public hospitals where the cost per patient per ICU day has been estimated at R22,700 [2]. Discovery Health, the largest private medical scheme in the country, at the time reported that the average cost of all COVID-19 hospital admissions across its members was R84,708. The average cost of an ICU admission for its members was substantially higher - at R169,525 - and these admissions were also reported to have the “highest variation in cost” [3]. While public sector ICU capacity was projected to be limited, overprovision of ICU beds in the private sector was a key finding of the recent Competition Commission Health Market Inquiry [4], presenting an opportunity for government to purchase additional services from private hospitals for use by public sector dependent patients.

There are a range of health care interventions to manage the progression of the COVID-19 pandemic. Amongst others, resources are required to carry out education, screening, testing, isolation and contact tracing programs, provision of personal protective equipment (PPE) to health workers, treatment in general/high care wards; and in the most critical cases, treatment in ICU. Given the expected downturn in an already weak economy [5] coupled with the increased demand for government resources for economic relief and other measures, the ability of government to commit additional funding to a resource-constrained public health sector is limited. Within this context, it is imperative that available government resources are used fairly and efficiently, and that the costs and effects of potential interventions and approaches to care are assessed and weighed against the opportunity costs of their required investment. Traditional economic evaluations can be time consuming with lengthy turnaround times. The rapid pace at which the pandemic unfolded and the imperative it created for policy decisions to be made quickly required the turnaround times for research and analysis informing policy to be shortened substantially. The objective of this study was to assess the cost-effectiveness of ICU management for admitted COVID-19 patients across the public and private health sector in South Africa using a “real time”, pragmatic and transparent health economic modelling approach. The results of this analysis can contribute towards evidence informed policy making and planning during COVID-19 surges when certain aspects of hospital capacity are expected to be breached.

## Methods

### Study design

MOSAIC, a health economic modelling collective established to respond to the need for prompt policy guidance for the South African response to COVID-19, carried out this cost-utility/effectiveness analysis of ICU care. The study was conducted using the principles of the International Decision Support Initiative Reference Case for economic evaluation [6]. It considers two generalised strategies for the inpatient management of severe and critical COVID-19 patients: *(1) general ward and ICU management (GW + ICU)*: admitted patients are managed in a combination of general wards and ICU, with escalation to ICU based on established clinical criteria for severity of disease; (*2) general ward management*: admitted patients are managed in general wards only. This approach enables an estimation of the added value of ICU care in comparison to a general ward only comparator. The latter is therefore a modelled “do nothing” strategy that provides estimates of the outcomes that could result from the severe rationing of ICU care during surges in COVID-19 cases. Costs are expressed as the cost per case admitted from the health systems perspective, in 2020 South African Rand (ZAR), including both public and private sector costs. Outcomes are expressed as disability adjusted life years (DALYs) and deaths. While the simple measures of “deaths avoided” or “lives saved” are useful and easy to interpret, they miss important treatment effects, such as improvements in morbidity, and they cannot be used to make comprehensive assessments of value for money compared to other treatment options in the health sector. In contrast, representing the impact of interventions as disability-adjusted life years (DALYs) averted allows consideration of health gains due to a reduction of both fatal and non-fatal disease burden; one DALY can be thought of as one lost year of healthy life. Incremental cost-effectiveness ratios (ICERs) are calculated as the difference in costs divided by the difference in health benefits of the treatment strategies, and are compared to a cost effectiveness threshold (CET) derived from an estimate of the marginal productivity of the public health system in South Africa [7]. If the ICER is lower than the CET then the marginal opportunity cost of the treatment strategy (in terms of lost health) is expected to be lower than the health benefits of the treatment strategy, indicating that the treatment strategy is likely to represent a cost-effective use of limited resources [6]. The time horizon for the analysis was from admission to discharge or death; while estimates of ongoing morbidity post discharge were included within DALYs, no costs after discharge were estimated. The years of life lost (YLL) from COVID-19 mortality was informed by a secondary actuarial analysis and was not discounted.

### Decision analytic model

**Fig. 1:**
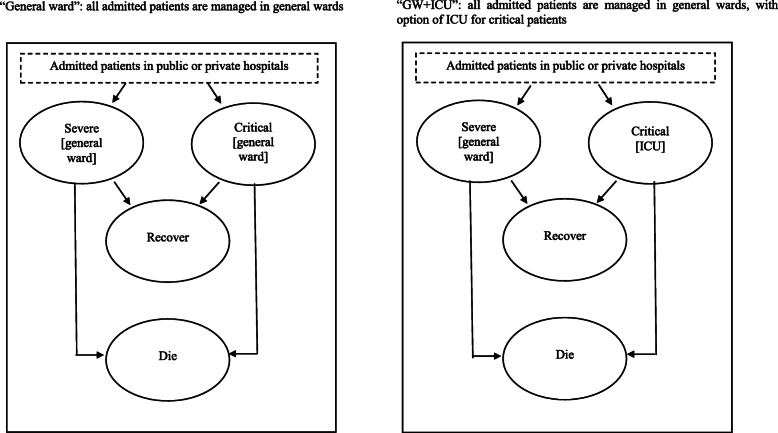
Decision analytic model structure

A decision analytic modelling framework was implemented in TreeAge Pro 2020 (TreeAge Software, Inc, Williams- town, Massachusetts, USA) and exported to Microsoft Excel for ease of stakeholder engagement and review, as depicted in Fig. 1. The model and accompanying user guide are available at 10.25375/uct.12382706.

Within the model, patients are ‘randomized’ to each treatment strategy (GW + ICU versus GW); and on admission (to public or private hospitals), patients are modelled as severe or critical. Depending on these factors, patients incur admission costs and accumulate health outcomes as they transition to one of two absorbing states: recover or die. Recovered patients receive a morbidity loss over the duration of their disease and thereafter are assumed to return to their pre-COVID-19 health state, while morbidity as well as YLLs are captured for those dying. Further details of these costs and outcomes are provided within Table 1.

**Table 1:** Costs and outcomes in each model state

Health state	Cost per health state	Transition outcome to recovered	Transition outcome to dead
Severe patients	Public / private sector cost per hospitalisation in general ward for severe patients	Disability weight for severe patients applied over duration of 1.5 months	Disability weight for severe patients applied over duration of 0.5 months; Years of Life Lost
Critical patients	Public / private sector cost per hospitalisation in general ward and ICU for critical patients (GW + ICU model) or general ward only (GW model)	Disability weight for critical patients applied over duration of 2 months	Disability weight for critical patients applied over duration of 0.5 months; Years of Life Lost

### Model variables

The model rests on 4 different types of variables: *mortality rates* based on severity of illness (i.e. severe versus critical) and approach towards disease management (i.e. GW + ICU versus GW); *utilisation* data including proportions of hospitalized individuals that are critical versus severe, proportion managed in public versus private hospitals and length of stay data for each patient type and management approach; *unit costs* per inpatient day in general wards and intensive care units specific to public and private hospitals; and *DALY* data including YLL, years lived with disability (YLD) and disability weights (DWs). A brief overview of each type of data is provided below. Evidence relating to disease progression and effectiveness of interventions has rapidly changed over the course of the COVID-19 pandemic. The parameters used in the model represent best available evidence as at end of May 2020.

#### Mortality rates

Mortality rates were extracted from the literature. A systematic search for articles published in English between 01/01/2019 and 30/05/2020 in Medline/PubMed was completed using the terms: “COVID-19” OR “novel coronavirus” OR “SARS-COV-2” OR COVID-19 OR 2019-COV OR “2019 novel coronavirus” AND “clinical characteristics” OR “clinical features” OR “clinical outcomes” AND “death” OR “mortality”. Additional relevant articles were sourced through a manual search of bibliographies of included articles.

**Fig. 2:**
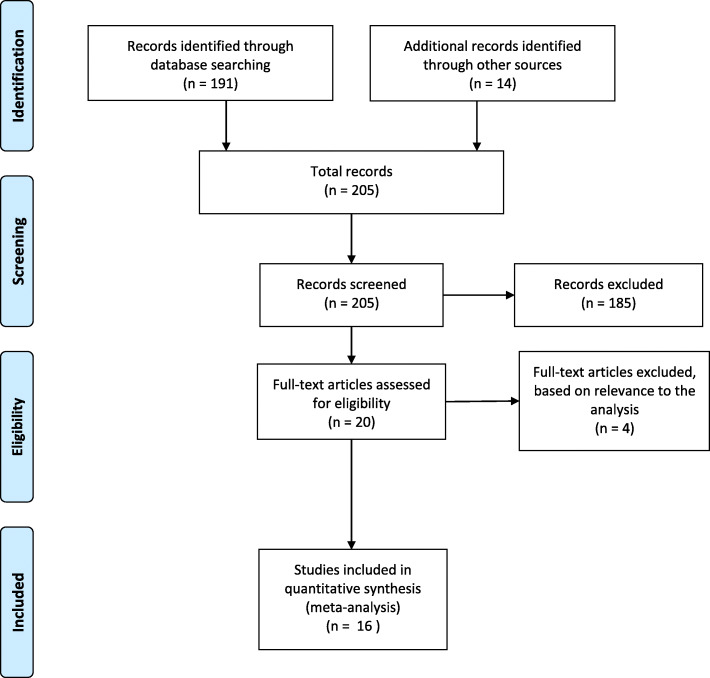
Flow diagram of search strategy and study selection

As outlined in Fig. 2, the results of the initial search were screened by title and abstract. The full texts of potentially relevant articles were retrieved and assessed for inclusion. When articles reported information from the same study sites but at two different time periods, only the articles with the updated statistics were included in this analysis. A total of sixteen observational studies (cross-sectional or cohort) and case series that reported the outcomes of hospitalized COVID-19 patients were included within quantitative synthesis. Average weighted estimates of the case fatality rate among ICU patients and non-ICU patients/patients dying in general ward were calculated using the formula: Deaths/(Deaths + Recovered).

#### Utilisation

Utilisation includes the proportions of hospitalized individuals that are critical versus severe and length of stay data for each patient type by type of management (utilization of ICU days by critical patients, utilization of general ward days for severe patients, and for critical patients before/after ICU). These variables were extracted from seven articles [8–16] identified in the above-mentioned systematic search. Average weighted estimates for each variable were calculated. Finally, the proportion of patients using public versus private hospitals was based on the proportion of South Africans with private medical scheme membership [17].

#### Unit costs

The model considers the costs of inpatient care in public and private hospitals through the inclusion of unit costs per general ward day and per ICU day. These are multiplied against the abovementioned length of stay estimates to generate a cost per admission. Private sector unit costs are based on the tariff rates in the “Guidelines on Public Private Collaboration in Response to COVID-19” published by the Department of Health [18]. Public sector unit costs were calculated using the Health Systems Trust District Health Barometer (12th Edition – 2016/17) datafile [19] which provides hospital-level estimates of expenditure per patient day equivalent (PDE) for all categories of public sector hospitals. These costs were inflated to 2020 prices using the Consumer Price Index [20] and a weighted average unit cost was calculated through weighting unit costs by the percentage of useable beds across levels of care. Because the HST-DHB data do not provide an estimate of the unit cost per ICU day, we estimated this by inflating the average weighted cost by the cost differential between ICU and general ward tariffs in the private sector.

#### DALYs

DALYs are calculated through the summation of YLL and YLD. YLL were informed by a South African actuarial analysis that utilised age- and co-morbidity adjusted mortality rates observed internationally and applied these to the South African population [21]. This resulted in an average estimate of 5.4 YLL per death in South Africa. A wide range on this parameter was tested in sensitivity analysis to reflect the relative uncertainty associated with transferring international mortality data to the South African context. At the time of analysis, duration of morbidity was unknown for COVID-19; assumptions were therefore made for these parameters. Disability weights for severe/critical COVID-19 patients were based on relevant estimates for similar conditions from the 2017 Global Burden of Disease study [22].

### Sensitivity analysis

Simple sensitivity analyses were run across all variables to assess the impact of changes on the ICER. Where possible, ranges for sensitivity analysis were based on upper and lower confidence intervals or interquartile ranges found within the systematic literature review. For the remaining variables, a 50 % increase/decrease was implemented, except for where this would move the variable out of feasible range (e.g. mortality rates can only fall within the range 0–1). Thereafter, threshold analyses were run to estimate the percentage change in variables that would render ICU cost-effective, using the published South African CET [7] as the cut-off for this determination. Finally, an additional scenario was modelled in order to incorporate the effect of administration of the steroid dexamethasone. This analysis entailed the inclusion of the cost of a course of dexamethasone (ZAR 160.85 for twenty 4 mg vials as per 2020 Essential Medicines List price), as well as rate ratio reductions in deaths from ICU (0.65) or from general wards (0.80) as provided in estimates from a United Kingdom based randomized controlled trial [23].

#### Ethical considerations

This is a modelled cost-effectiveness/utility analysis using published secondary data; no ethical approval was therefore required.

## Results

### Model variables

Table 2 provides a summary of the variables used in the model, together with the ranges on variables used for sensitivity analysis. As is shown, the unit cost per inpatient day in GW was estimated at approximately ZAR 3,700 in public hospitals versus ZAR 5,300 in private; while ICU care was estimated at approximately ZAR 18,000 and ZAR 25,000 in public versus private respectively. In terms of utilisation, the literature review provided an estimate of 21.25 inpatient days in general wards for severe patients; 1 day in general ward plus 8.8 days in ICU for critical patients; and that 21 % of admitted patients would be critical and in need of ICU. Mortality estimates suggested that 11 % of severe patients treated in general wards and 54 % of critical patients treated in ICU would die.

**Table 2:** Summary of model variables

**Description**	**Base Value**	**Low Value**	**High Value**	**Method or assumption (range for sensitivity analysis)**	**Reference or source**
**Unit costs**					
Cost per general ward day public	3,727.35	1,863.68	5,591.03	Average weighted expenditure per patient day equivalent (± 50 %)	[19]
Cost per ICU day public	17,844.88	8,922.44	26,767.31	Average weighted expenditure per patient day equivalent inflated using private tariff differential (± 50 %)	Assumption
Tariff per general ward day private	5,251.66	2,625.83	7,877.48	Published tariff rates (± 50 %)	[18]
Tariff per ICU day private	25,142.55	12,571.28	37,713.83	Published tariff rates (± 50 %)	[18]
**Utilisation**					
LoS in general ward in severe patients	21.25	7.25	43.00	Literature review (IQR)	[11, 12]
LoS in ICU in critical patients	8.80	4.30	13.30	Literature review (IQR)	[8–10, 13, 14]
LoS in general ward in critical patients in absence of ICU	8.80	4.30	13.30	Assumed to be the same as critical patients treated in ICU	Assumption
LoS in general ward in critical patients before/after ICU	1.00	0.00	3.00	Literature review (IQR)	[12]
Proportion needing ICU	0.21	0.05	0.50	Literature review (low and high value)	[12–15, 24–27]
Proportion reliant on public health system	0.83	0.42	1.00	Percentage of population without Medical Scheme coverage (-50 %;1)	[28]
**Mortality rates**					
Proportion of severe patients dying	0.11	0.00	0.13	Literature review (low and high value)	[12, 14–16, 24]
Proportion of critical patients dying from ICU	0.54	0.24	0.88	Literature review (low and high value)	[8–10, 12–16, 24, 29, 30]
Proportion of critical patients dying without access to ICU	0.88	0.70	1.00	Assumed high value on critical patients dying from ICU (-20 %;1)	Assumption
**DALYs**					
Disability weight in severe patients	0.13	0.09	0.19	Disability weight for severe lower respiratory tract infection (95 % CI)	[22]
Disability weight in critical patients	0.41	0.27	0.56	Disability weight for severe pneumoconiosis (95 % CI)	[22]
Duration of illness in severe patients	0.13	0.06	0.19	1.5 months (± 50 %)	Assumption
Duration of illness in critical patients	0.17	0.08	0.25	2 months (± 50 %)	Assumption
Duration of illness prior to death	0.04	0.02	0.06	0.5 months (± 50 %)	Assumption
Years of life lost if dying from COVID-19	5.40	2.70	8.10	5.4 years (± 50 %)	[21]
**Other**					
Cost-effectiveness threshold per DALY averted	38,465.46			Used to assess value for money; if ICER < CET intervention potentially cost-effective	[7]

As highlighted in the table, there are two key unknowns for this economic evaluation that both apply to the GW strategy. The first is the proportion of critical patients that would die if they did not have access to ICU. This unknown has been based on the high value found in the meta-analysis for critical patients dying from ICU (88 % mortality) with ranges for sensitivity analysis including 70 % and 100 % mortality. The other unknown is the length of stay for these critical patients, which is assumed to be the same as for critical patients managed in ICU.

Other important unknowns include disability weights for COVID-19, with the DW for severe lower respiratory tract infection assumed for severe COVID-19 patients and the DW for severe pneumoconiosis assumed for critical patients, as extracted from the Global Burden of Disease study [22]. Duration of illness is assumed to be 0.5 months in those dying, 1.5 months in severe patients and 2 months in critical patients. Finally, YLL is based on international mortality rates per 100 000 population by age and co-morbidity applied to the South African demographic structure [21].

### Cost-effectiveness

Table 3 summarizes the cost-effectiveness results. Assuming base case values across all variables, the model produces a cost per admission of ZAR 75 127 versus ZAR 103 030, deaths at 27 % versus 20 %, and DALYs at 1.48 versus 1.10 in the general ward versus GW + ICU strategy, respectively. The incremental cost-effectiveness ratio is ZAR 73 091 per DALY averted or ZAR 390 798 per death averted. Taking into account the proportions of admitted patients who are severe versus critical, the general ward strategy requires 18.85 general ward days per admission, while the GW + ICU strategy requires 17 general ward days and 1.85 ICU days.

**Table 3:** Base case cost-effectiveness results (2020 ZAR)

**Strategy**	**Cost (ZAR)**	**DALYs**	**Deaths**	**ICU Days**	**Genl. Ward Days**	**Incr. Cost per DALY Averted**	**Incr. Cost per Death Averted**
Genl. Ward	75 127	1.48	0.27	-	18.85		
Genl. Ward + ICU	103 030	1.10	0.20	1.85	17.00	73 091	390 798

*DALYs* Disability adjusted life years, *Genl.* General, *ICU* Intensive care unit, *Incr.* Incremental

### Sensitivity analysis

**Fig. 3:**
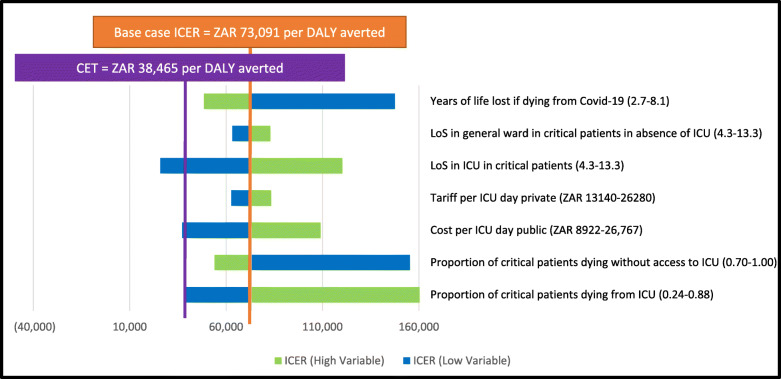
Tornado diagram summarizing simple sensitivity analyses

Sensitivity analyses were run on all variables with the ranges for these analyses presented in Table 2. The tornado diagram in Fig. 3 summarizes the results for the seven analyses that generated the largest changes to the ICER. All other analyses generated lower or negligible changes in cost-effectiveness. To guide an interpretation of these findings, ICERs are compared against the cost-effectiveness threshold of ZAR 38,465 per DALY averted [7]. This represents the marginal productivity of the South African health system; interventions would need to generate an ICER lower than or equivalent to this value to be potentially cost-effective.

As is shown in Fig. 3, three analyses showed promise for generating a cost-effective ICER for the GW + ICU strategy: (1) length of stay in ICU in critical patients (shorter LoS improves cost-effectiveness); (2) cost per ICU day (less costly services improves cost-effectiveness); and (3) proportion of critical patients dying from ICU (lower mortality improves cost-effectiveness). Other important drivers of cost-effectiveness include YLL (if more lost life years can be averted, ICU becomes more cost-effective) and the outcomes of critical patients managed in general wards (poorer outcomes improves the relative benefits of ICU). In effect, these analyses indicate that if ICU were less costly (either through reduction in the unit cost or the length of stay) or more effective (either through reduced mortality or targeting the service towards patients with higher potential years of life to lose), purchasing additional intensive care from private hospitals would be more likely to be a cost-effective use of government resources.

The threshold analysis (Table 4) takes this one step further to estimate the exact percent change in variables needed to generate cost-effective ICERs. As is shown, five threshold values were found. To render additional purchasing of ICU cost-effective, we would need a 57 % decrease in the proportion of critical patients dying from ICU; 48 % decrease in the public sector cost per ICU day; 38 % decrease in ICU length of stay; 179 % increase in LoS in critical patients managed in general wards; or 89 % increase in YLL in those dying from COVID-19.

**Table 4:** Threshold analyses

**Variable Description**	**Base Value**	**Threshold Value**	**% Change**	**Interpretation**
Proportion of critical patients dying from ICU (pdeadICU)	0.54	0.23	-57 %	57 % decrease in ICU mortality renders ICU cost-effective
Cost per ICU day public (cICUpub)	17,845	9,227	-48 %	48 % decrease in cost per ICU day in public sector renders ICU cost-effective
LoS in ICU in critical patients (uICU)	8.80	5.5	-38 %	38 % decrease in ICU length of stay renders ICU cost-effective
LoS in general ward in critical patients in absence of ICU (uIPDcritical)	8.80	24.59	179 %	179 % increase in length of stay for critical patients managed in general ward renders ICU cost-effective
Years of life lost if dying from COVID-19 (YLL)	5.40	10.21	89 %	89 % increase in YLL in those dying renders ICU cost-effective

Finally, we conducted a scenario analysis on the impact of dexamethasone on the cost-effectiveness of ICU through the inclusion of the price of a course of dexamethasone and rate ratio reductions in mortality as provided from a UK based randomized control trial [23]. The results from this scenario indicate a slightly improved ICER of ZAR 70,400 per DALY averted for the GW + ICU strategy.

## Discussion

Based on the evidence available at the time of analysis (up to end of May 2020) and at base case values, this article presents the cost-effectiveness of alternative management strategies for admitted COVID-19 patients in South African hospitals. The policy relevance of these findings relates to decision-making during surges in COVID-19 cases when hospital capacity becomes constrained and hard choices need to be made around the rationing of care. During these surges, this analysis suggests that it may not be cost-effective for government to purchase additional ICU beds from private hospitals. In addition, if public sector resources (such as staffing) are severely constrained due to surges in COVID-19, the analysis suggests that better returns will be achieved through the provision of additional staffed and operational general ward beds within public hospitals. As shown in sensitivity analyses, these findings are driven by three key factors: (1) the high mortality in critical patients admitted to ICU; (2) the relatively low number of years of life lost in those dying; and (3) the relatively high cost of ICU services (which is a function of unit cost and length of stay).

While intensive care is expensive, if it were effective at preventing deaths in critically ill COVID-19 patients, it would have generated more favourable results. However, our meta-analysis of evidence from nine available studies suggested that outcomes were poor [8–16, 24, 29, 30]. The mortality rate for critically ill patients managed in ICU was on average 54 % (range: 28 %-88 %) meaning that for a substantial proportion of critical COVID-19 patients, admission to ICU was unlikely to be a life-saving intervention. While the inclusion of dexamethasone improved the ICER results, it did not move the ICER into the cost-effective range. That said, the dexamethasone scenario illustrates how the inclusion of new technologies such as medicines could generate changes in the economics of inpatient care for COVID-19.

Approaches to decision making in the face of resource scarcities (also called “priority setting”) commonly includes four fundamental values or ethical principles drawn from theories of distributive justice [31, 32]: (1) “utilitarianism” - doing the greatest good for the greatest number of people, either by saving the highest number of lives and/or saving the largest number of life-years, which is the basis of cost-effectiveness; (2) “egalitarianism” – providing equal access or equal treatment for equal need; (3) “rule of rescue” – providing urgent, life-saving treatments irrespective of the cost; and (4) “desert” - promoting and rewarding social usefulness [33]. Based on utilitarian or egalitarian approaches, there would be limited merit in government purchasing additional ICU capacity from private hospitals. However, justification for investing in ICU may be found through the application of other ethical principles such as the “rule of rescue”. The latter calls on society to respond to the extreme risk faced by an identifiable individual, however it generally only holds when the numbers are small. The difficulty to face with COVID-19 is that while many of us align with a rule of rescue based response, the stark realities of resource constraints call on us to acknowledge that the allocation of government resources to purchase ICU capacity from private hospitals would have a high opportunity cost in terms of the competing uses of those funds.

While the economic evaluation methodology can provide quantitative evidence to inform each of these ethical principles, and hence is a key input to any consideration of distributive justice [34], experience globally suggests that a multi-ethical framework is more likely to result in a fairer allocation of resources. Moreover, because reasonable people should be expected to disagree on the relative merits of these ethical principles, priority setting needs to be vested within procedural justice, a key aspect of which is transparent deliberation [35]. Our real-time and open access approach to modelling provides one example of how such transparency can be facilitated.

There are a number of limitations to this study and similar studies. The urgency to inform decision making and restrictions on primary data collection necessitated a reliance on secondary data while the ongoing emergence of new information required flexibility in model building. To address these concerns (1) a comprehensive systematic review was carried out to ensure that all of the available information was fed into the model and (2) an open access modelling framework [36] with a user guide [37] was developed to facilitate full exploration of uncertainty through sensitivity analysis and to allow for parameters to be quickly and easily updated as new information becomes available. A further limitation is that this analysis is static in nature and does not provide projections of the total need for ICU or general ward beds as the epidemic unfolds across South Africa during 2020 and 2021. The implication is that the findings are of relevance during surges in COVID-19 when capacity is breached, and hard choices need to be made regarding the rationing of both general ward and ICU care. The findings do not necessarily apply to COVID-19 ICU care during times when COVID-19 admissions are manageable.

## Conclusions

This rapid analysis provided key evidence on the cost-effectiveness of alternative management strategies in the hospital care of critical and severe patients with COVID-19 disease in the South African setting. It has shown that ICU use for COVID-19 patients was unlikely to be cost-effective on the margin, and therefore an expansion of ICU capacity during COVID-19 surges through government purchase of private services for use by public sector patients (at current prices and evidence of effectiveness) may not be the best use of limited health resources, whether from utilitarian or egalitarian ethical perspectives.

There are few (if any) examples of decision analytic modelling and cost effectiveness analysis being conducted in “real time” to inform policy decisions in the South African public health sector. The rapid, pragmatic and transparent analysis employed by the MOSAIC group demonstrates a potential approach for further evidence generation for decisions relating to the COVID-19 pandemic response and South Africa’s wider priority setting agenda.

## Data Availability

The model, associated data and user guide are available online at Zivahub: doi:10.25375/uct.12382706.

## Abbreviations

CET: Cost-effectiveness threshold
DW: Disability weight
DALYs: Disability adjusted life years
GW + ICU: General ward plus intensive care unit
ICER: Incremental cost-effectiveness ratio
ICU: Intensive care unit
PDE: Patient day equivalent
YLL: Years of life lost
YLD: Years lived with disability
